# Short-Term Mortality Rates during a Decade of Improved Air Quality in Erfurt, Germany

**DOI:** 10.1289/ehp.11711

**Published:** 2008-10-07

**Authors:** Susanne Breitner, Matthias Stölzel, Josef Cyrys, Mike Pitz, Gabriele Wölke, Wolfgang Kreyling, Helmut Küchenhoff, Joachim Heinrich, H.-Erich Wichmann, Annette Peters

**Affiliations:** 1 Institute of Epidemiology and; 2 Focus Network Nanoparticles and Health (NanoHealth), Helmholtz Zentrum München–German Research Center for Environmental Health, Neuherberg, Germany;; 3 Environment Science Center, University of Augsburg, Augsburg, Germany;; 4 Institute of Inhalation Biology, Helmholtz Zentrum München–German Research Center for Environmental Health, Neuherberg, Germany;; 5 Department of Statistics and; 6 IBE-Chair of Epidemiology, Ludwig-Maximilians-University, Munich, Germany

**Keywords:** accountability research, air pollution, improved air quality, mortality, particulate matter, ultrafine particles

## Abstract

**Background:**

Numerous studies have shown associations between ambient air pollution and daily mortality.

**Objectives:**

Our goal was to investigate the association of ambient air pollution and daily mortality in Erfurt, Germany, over a 10.5-year period after the German unification, when air quality improved.

**Methods:**

We obtained daily mortality counts and data on mass concentrations of particulate matter (PM) < 10 μm in aerodynamic diameter (PM_10_), gaseous pollutants, and meteorology in Erfurt between October 1991 and March 2002. We obtained ultrafine particle number concentrations (UFP) and mass concentrations of PM < 2.5 μm in aerodynamic diameter (PM_2.5_) from September 1995 to March 2002. We analyzed the data using semiparametric Poisson regression models adjusting for trend, seasonality, influenza epidemics, day of the week, and meteorology. We evaluated cumulative associations between air pollution and mortality using polynomial distributed lag (PDL) models and multiday moving averages of air pollutants. We evaluated changes in the associations over time in time-varying coefficient models.

**Results:**

Air pollution concentrations decreased over the study period. Cumulative exposure to UFP was associated with increased mortality. An interquartile range (IQR) increase in the 15-day cumulative mean UFP of 7,649 cm^−3^ was associated with a relative risk (RR) of 1.060 [95% confidence interval (CI), 1.008–1.114] for PDL models and an RR/IQR of 1.055 (95% CI, 1.011–1.101) for moving averages. RRs decreased from the mid-1990s to the late 1990s.

**Conclusion:**

Results indicate an elevated mortality risk from short-term exposure to UFP. They further suggest that RRs for short-term associations of air pollution decreased as pollution control measures were implemented in Eastern Germany.

Ambient concentrations of particulate matter (PM) have been consistently associated with daily mortality (Analitis et al. 2006; Dominici et al. 2005; Health Effects Institute 2003; Pope and Dockery 2006; Zanobetti et al. 2003). Associations between ambient concentrations of nitrogen dioxide or carbon monoxide and daily mortality have been observed (Samoli et al. 2006, 2007), but the causality of the NO_2_ effects is being debated [World Health Organization (WHO) Europe 2006].

In recent years, air pollution concentrations have been reduced by emission controls and fuel replacement. Accountability of these measures is of major concern for the regulating agencies as well as for the regulated entities. This is of particular interest because some areas in the United States and in Europe are still out of compliance with enacted standards (U.S. Environmental Protection Agency 2004) or suggested guideline values (WHO Europe 2006).

A small number of studies have been conducted that fit within this research framework. Examples are studies on the banning of coal sales in Dublin, Ireland (Clancy et al. 2002), the reduction of sulfur in fuels in Hong Kong, China (Hedley et al. 2002), and traffic restrictions during the 1996 Olympic Games in Atlanta, Georgia, USA (Friedman et al. 2001) and the 2002 Asian Games in Busan, Korea (Lee et al. 2007). A recent analysis of data from the Harvard Six Cities study investigated whether a decline in mortality rates is largest in cities with the largest reduction in long-term average PM (Laden et al. 2006). Lately, a study investigated the association between particulate air pollution and mortality in the United States during a period when several key particulate-related air pollution control programs were implemented (Dominici et al. 2007).

Political changes in Central and Eastern Europe have resulted in the restructuring of the Eastern bloc industries, improved emission controls, and a changed car fleet (Acker et al. 1998). The improved emission control led to a complete fuel replacement and an exchange of brown coal for natural gas in power plants and in domestic heating. All those changes resulted in improved air quality in this region within a decade (Ebelt et al. 2001) and have provided an opportunity for a natural experiment to evaluate the health impacts of air pollution. Heinrich et al. (2002) assessed the impact of declines of total suspended particulates and sulfur dioxide in Eastern Germany in the 1990s on the prevalence of nonallergic respiratory disorders in children.

We designed the present study to investigate the associations between selected criteria pollutants [NO_2_, CO, and PM < 10 μm (PM_10_) and PM < 2.5 μm (PM_2.5_) in aerodynamic diameter], ultrafine particle number concentrations (UFP), and daily mortality over a 10.5-year period after the German unification, with a particular emphasis on changes in relative risks (RRs) for daily mortality in association with these pollutants, as they changed during the study period.

## Material and Methods

### Study area and period

We conducted the study in Erfurt, the capital of the state of Thuringia, Germany, from 1 October 1991 to 31 March 2002. The city of Erfurt has a population size of approximately 200,000 inhabitants and is surrounded by mountain ridges of 100–150 m on three sides and high-rise buildings on the fourth side. Because of an administrative reform in 1994, a number of autonomous communities were incorporated into the city area of Erfurt [see Supplemental Material, Figure 1 (http://www.ehponline.org/members/2008/11711/suppl.pdf)]. The city area including those communities measures 21 km from north to south and 22.4 km from east to west. However, about 90% of the inhabitants of Erfurt live within a rectangular area of 5 km × 3 km around the old city center.

### Mortality data

We obtained copies of death certificates without the name and address of the decedents from local health authorities to comply with the rules of the German data privacy law. We excluded deaths of infants (< 1 year of age) and non-natural deaths [*International Classification of Diseases, 9th Revision* (ICD-9; WHO 1975) codes ≥ 800 and *10th Revision* ICD-10 (WHO 1993) codes ≥ S00]. Data collection methods and quality control mechanisms are described elsewhere (Wichmann et al. 2000).

For the analysis of air pollutants, for which data were available from 1991 onward, we considered deaths occurring within the old city limits of Erfurt [see Supplemental Material, Figure 1 (http://www.ehponline.org/members/2008/11711/suppl.pdf)]. For the analysis of air pollutants in the period 1995–2002, we also included deaths in the incorporated communities. This strategy provides estimates comparable with earlier analyses of subsets of the data set presented here (Stölzel et al. 2003, 2007; Wichmann et al. 2000).

### Air pollution and meteorologic data

We obtained daily mean concentrations of NO_2_ and CO from a state-run network monitoring station for the entire study period. During the winter of 1991–1992 and from September 1995 onward, we sampled the particle size distribution at a research monitoring site located around 1 km south of the city center [see Supplemental Material, Figure 1 (http://www.ehponline.org/members/2008/11711/suppl.pdf)]. The measurement station can be classified as an urban background site and had a distance of 40 m from the nearest major road. We measured size-specific particle number concentrations by an aerosol spectrometer as described elsewhere (Pitz et al. 2001; Tuch et al. 2003; Wichmann et al. 2000). For the present analysis, we computed daily means of UFP (size range, 0.01–0.1 μm) from the spectra. We obtained data for the number concentrations (NC) for three specific size ranges—0.01–0.03 μm, 0.03–0.05 μm, and 0.05–0.1 μm—for September 1995 to August 2001. We computed daily means of PM_2.5_ assuming spherical particles of a mean density of 1.53 g/cm^3^ (Pitz et al. 2001; Wichmann et al. 2000). Additionally, we collected PM_10_ on a Harvard Impactor (Air Diagnostics and Engineering Inc., Harrison, ME, USA).

We imputed missing values in the UFP, PM_2.5_, and PM_10_ time series using concurrent measurements. A detailed description of the imputation process can be found elsewhere (Peters et al. in press; Stölzel et al. 2007). Between 1 April 1994 and 1 February 1995, the NO_2_ concentrations were unusually low and exhibited very little variation. Therefore, we excluded this period from the analyses.

We obtained daily mean air temperature and relative humidity from a site of the German Meteorologic Service located at Erfurt Airport 5 km west of the measurement station.

We calculated exposure lags up to 14 days for the air pollution data. In addition, we calculated the means of lags 0–5 and 0–14 for the air pollution data and the means of lags 0–1, 0–2, and 0–5 for the meteorologic variables, if at least half of the relevant lags were available.

### Other data

We obtained data on influenza epidemics from the Arbeitsgemeinschaft Influenza [AGI (German Influenza Working Group) 2003] in the form of a weekly doctor’s practice index for each winter season (October through April). This index indicates the relative deviation of the number of doctor visits because of acute respiratory symptoms compared with a background level averaged for the whole of Germany.

### Statistical analysis. Statistical model

We analyzed data using generalized semiparametric Poisson regression models. We used natural cubic and penalized regression splines to allow for nonlinear confounding effects. We considered constant as well as time-varying associations between pollutants and daily mortality.

We built confounder models separately for the two analysis periods, 1991–2002 (gaseous pollutants and PM_10_) and 1995–2002 (UFP and PM_2.5_), without including any air pollutants. As potential confounders, we considered a global trend over calendar time, seasonal and weekday variations, influenza epidemics, and air temperature and relative humidity. We selected models by minimizing Akaike’s Information Criterion (Akaike 1973) and the absolute value of the sum of the partial autocorrelation function (Touloumi et al. 2006). To ensure sufficient adjustment for season and meteorology, we forced long-term time trend and same-day air temperature into all models. We considered lags 0–2, the mean of lags 0–1, and the mean of lags 0–2 for the weather variables; for the doctor’s practice indexes, we assessed shifts of up to ± 3 weeks, because the influenza epidemics may have reached their peaks in Erfurt at another time than in the whole of Germany.

In the final confounder models [see Supplemental Material, Table 1 (http://www.ehponline.org/members/2008/11711/suppl.pdf)], we readjusted the number of degrees of freedom (df) for the smooth function of time trend, because many of the meteorologic variables exhibit seasonal patterns themselves and hence capture part of the observed seasonal trends in the outcome (Touloumi et al. 2004).

In the last step of the analysis, we added air pollutants separately to the models and estimated associations linearly. To investigate cumulative associations between air pollutants and daily mortality counts up to 14 days after exposure, we used polynomial distributed lag (PDL) models (Schwartz 2000; Zanobetti et al. 2003). We constrained the lag coefficients to follow a third-degree polynomial of the lag number. We obtained cumulative estimates as the sum of the estimated coefficients for any given lags in the PDL models. Additionally, we investigated the associations between 6-day or 15-day averages of the pollutants and mortality.

For estimating pollution–mortality associations over different periods, we replaced the assumption of a time-constant overall pollution effect by using an interaction term approach from which the air pollutant has been removed as a main effect. This means that we included indicator variables for the periods and multiplicative linear terms of the pollutant and the period indicators. This approach can be seen as a simple time-varying coefficient model that results in a step function or as a nested approach to investigating effect modification by period (Van Ness and Allore 2006). We used a likelihood ratio test to determine whether there were indeed differences between periods.

Alternatively, we adapted statistical tools developed by Brezger and Lang (2006) for Bayesian varying coefficient models. Specifically, we estimated time-varying associations of the pollutants by modeling the effect estimate as a smooth function of time trend β_poll_ = *f*_poll_(*t*). The smooth effect *f*_poll_(*t*) was modeled using a Bayesian adaptation of penalized B-splines (Brezger and Lang 2006). A more detailed description of this modeling approach can be found elsewhere (Peters et al. in press); for a similar approach, see Lee and Shaddick (2007).

We analyzed data using the package “mgcv” in the statistical software R (R Development Core Team 2003) and using BayesX (Brezger et al. 2005). We present effect estimates as RRs for mortality together with 95% confidence/credible intervals (CIs) based on an increase in air pollution concentrations from the first to the third quartile [interquartile range (IQR)].

### Sensitivity analyses

To explore the robustness of the models, we performed sensitivity analyses using different values of smoothness for the functions of time trend and air temperature. Furthermore, we used a categorical variable for all days of the week instead of a Sunday indicator. For the analysis period 1995–2002, we performed a sensitivity analysis using the same confounder model as for period 1991–2002. Moreover, we investigated the association between daily mortality and UFP adjusting for other pollutants in two-pollutant models. We finally investigated the exposure–response relationship between air pollutants and mortality. We replaced the linear term of the pollutant concentrations with a fixed 3-df regression spline. We used a likelihood ratio test with 2 df that compares the original main model with the smoothed model, and visual inspection to assess whether the smoothed exposure–response curve resembles a straight line. Additionally, we compared different values of smoothness by the generalized cross-validation score as provided in R.

## Results

### Mortality data

We collected 17,713 death certificates that met the inclusion criteria. On average, we observed 4.6 deaths per day. Around 10% of all cases occurred in the outlying communities that were incorporated in 1994 [see Supplemental Material, Figure 1 (http://www.ehponline.org/members/2008/11711/suppl.pdf)].

### Air pollutants and meteorologic data

**Figure 1:**
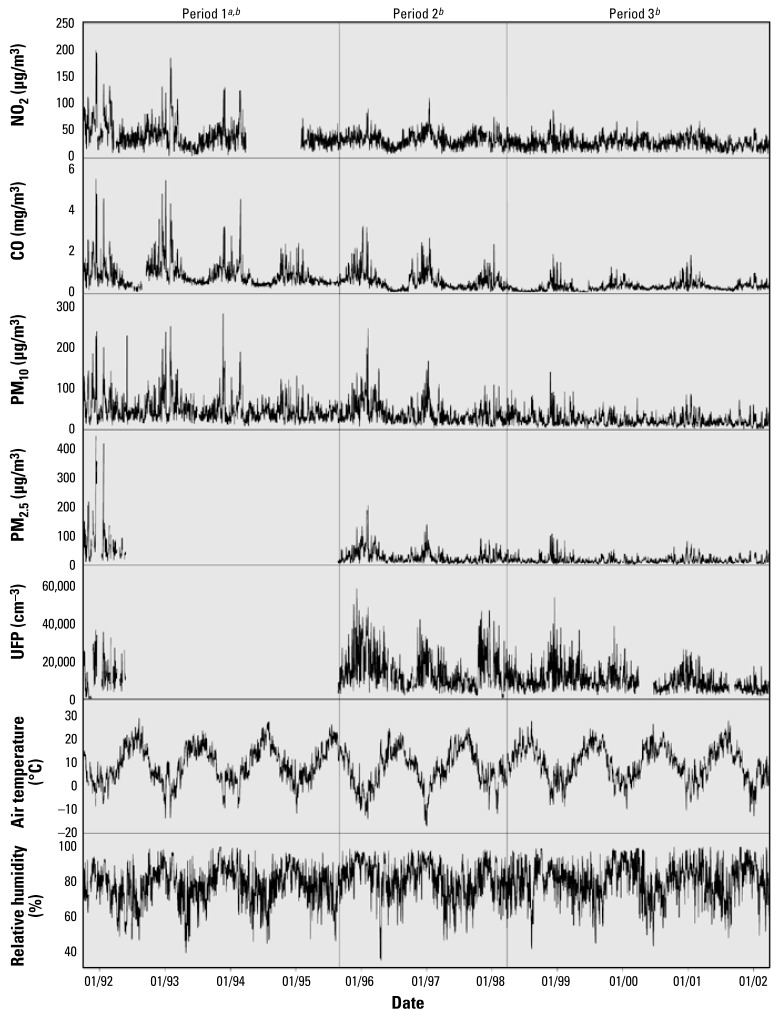
Daily average concentrations of air pollutants and meteorologic data in Erfurt, Germany, between 1991 and 2002. ***a***Plausible data only; data from 1 April 1994 to 31 January 1995 omitted. ***b***Imputed time series.

There were significant changes in air pollution concentrations during the 10.5 years of observation. We therefore divided the full study period into three smaller periods: period 1, 1 October 1991 to 31 August 1995; period 2, 1 September 1995 to 28 February 1998; and period 3, 1 March 1998 to 31 March 2002 (Figure 1).

NO_2_, CO, PM_2.5_, and PM10 levels decreased continuously between period 1 and period 3 (Figure 1, Table 1). However, PM_2.5_ and UFP were measured only during the first winter of period 1. During period 2, the UFP remained stable and decreased only after 1999—that is, in the middle of period 3. All pollutants exhibited a pronounced seasonal pattern, with higher concentrations during the winters and lower concentrations during summers (Figure 1). UFP was only moderately correlated with PM_10_ and PM_2.5_ (Spearman rank correlation = 0.57 and 0.48), whereas PM_10_ and PM_2.5_ were highly correlated [Spearman rank correlation = 0.85; see also Supplemental Material, Table 2 (http://www.ehponline.org/members/2008/11711/suppl.pdf)].

### Regression results

Regression results showed an association between daily mortality and UFP (Tables 2 and 3). We observed the strongest associations for cumulative exposures up to 14 days [Table 3; see also Supplemental Material, Figures 2 and 3 (http://www.ehponline.org/members/2008/11711/suppl.pdf)]. Smaller size ranges of UFP showed larger associations, although the RRs per IQR increase were not significant (Tables 2 and 3). We observed no associations for PM_2.5_ and PM_10_. Risk estimates obtained for deaths occurring in the old city limits were stronger for the period 1995–2002. RRs per IQR increase of single lags up to a lag of 5 days are shown in Supplemental Material, Table 3 (http://www.ehponline.org/members/2008/11711/suppl.pdf).

**Figure 2:**
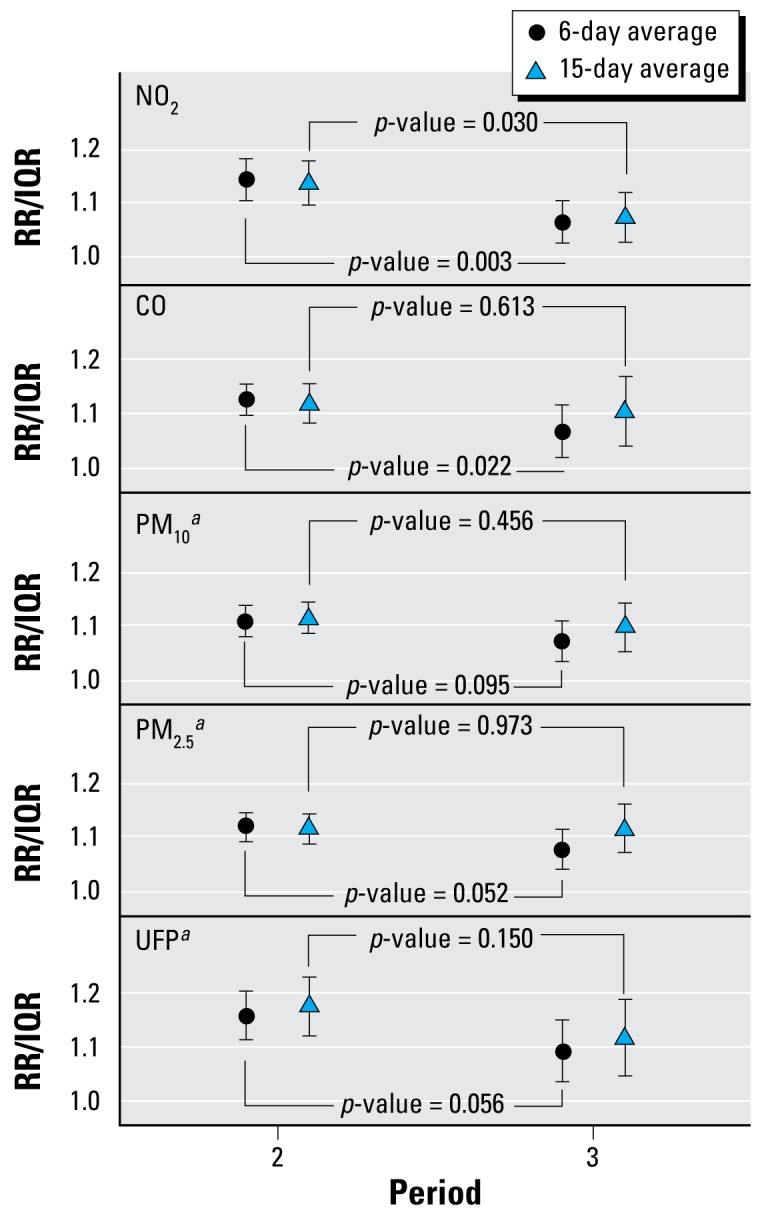
Cumulative RR of mortality (together with 95% CI) for different periods in association with NO_2_, CO, PM_10_, PM_2.5_, and UFP in Erfurt estimatedusing 6-day (circles) and 15-day (triangles) averages. Periods: period 2, 1 September 1995 to 28 February 1998; and period 3, 1 March 1998 to 31 March 2002. Overall IQR values: for the 6-day average: NO_2_, 12.7 μg/m^3^; CO, 0.31 mg/m^3^; PM_10_, 17.2 μg/m^3^; PM_2.5_, 13.3 μg/m^3^; and UFP 8,439 cm^−3^; for the 15-day average: NO_2_, 11.0 μg/m^3^; CO, 0.31 mg/m^3^; PM_10_, 14.5 μg/m^3^; PM_2.5_, 11.5 μg/m^3^; and UFP 7,649 cm^−3^. Cumulative RRs were estimated for mortality within new city limits. *^a^*Imputed time series.

Figure 2 and Table 4 present cumulative RRs estimated for different periods. The associations between air pollutant concentrations and mortality were strongest for the years 1995 to 1998 and decreased afterward (Figure 2). Although gaseous pollutant concentrations generally did not exhibit strong associations with mortality (Tables 2 and 3), they also showed adverse effects during 1995–1998 (Table 4, Figure 2). Using the alternative approach with a smooth function to model time-varying associations produced estimates that are comparable in shape [see Supplemental Material, Figure 4 (http://www.ehponline.org/members/2008/11711/suppl.pdf)].

### Sensitivity analyses

We performed a number of sensitivity analyses (Table 5). Further reducing the df for the spline to model trend and seasonality did not change or only slightly decreased the risk estimates for UFP. Using a temperature model with an increased df did not change the results for the cumulative 6-day RR of UFP and only slightly increased the results for all other measures. Using an indicator variable for all days of the week instead of the Sunday indicator only slightly changed the risk estimates. Using the same confounder model as for the period 1991–2002 resulted in quite comparable cumulative RRs. Results of the sensitivity analyses for the ultrafine size range NC 0.03–0.05 μm are shown in Supplemental Material, Table 4 (http://www.ehponline.org/members/2008/11711/suppl.pdf). Adjusting for other pollutants in two-pollutant models also increased the RRs for mortality in association with UFP [see Supplemental Material, Table 5 (http://www.ehponline.org/members/2008/11711/suppl.pdf)]. Exposure–response relationships between UFP or the ultrafine size range NC 0.03–0.05 μm and mortality [see Supplemental Material, Figure 5 (http://www.ehponline.org/members/2008/11711/suppl.pdf)] and corresponding likelihood ratio tests (data not shown) indicated a linear exposure–response relationship.

## Discussion

Economic and political changes and the adoption of new technologies in Eastern Germany have resulted in clear improvements in ambient air quality. We observed the largest RRs for UFP. We further observed that RRs varied over time for some of the pollutants, which could not be explained by nonlinearity in the exposure–response functions. We found no significant associations for PM_2.5_ and PM_10_. In general, associations between mortality and air pollution were lower at the end of the study period than during the 1990s. We observed the strongest associations in the transition period 1995–1998, when changes in source characteristics took place and the benefits of ambient air quality were not yet completely achieved.

Previous studies have indicated an association between UFP and daily mortality, but evidence is limited. Forastiere et al. (2005) showed a significant increase of out-of-hospital coronary deaths in Rome, Italy, in association with an increase in same-day UFP. Previous analyses of data from Erfurt, Germany, for the periods 1995–1998 and 1995–2001 indicated delayed associations between UFP and daily mortality (Stölzel et al. 2007; Wichmann et al. 2000). We observed the highest association between UFP and daily mortality with a lag of 4 days. This analysis showed that the RR estimates for the entire period were somewhat smaller than in the previous analyses for data of the years 1995–1998, which is consistent with the results of the time-varying analyses. The analyses presented in this study showed largest RRs between 1995 and 1998, which, by chance, was about the time period of the initial mortality study in Erfurt (Stölzel et al. 2003; Wichmann et al. 2000). UFP and a UFP subclass showed evidence for linear exposure–response relationships when we applied smoothing techniques, which adds to the consistency of the associations. Two-pollutant models suggested that the observed association of UFP with mortality was not confounded by other pollutants.

The association between PM and all-cause mortality has been consistently observed (Analitis et al., 2006; Dominici et al. 2005; Health Effects Institute 2003). Large multi-center studies in Europe and the United States have reported effect estimates of 0.2–0.6% per 10-μg/m^3^ increase of PM_10_ (Health Effects Institute 2003). Previous analyses of data from Erfurt pointed to an association of mortality with fine PM mass (Wichmann et al. 2000). A 19.9-μg/m^3^ increase in the PM_2.5_ concentration was significantly associated with 3.0% more daily deaths (same day). In the present study, we observed no significant associations for PM_10_ or PM_2.5_. However, time-varying models indicated positive effect estimates for the period 1995–1998, whereas in the other periods the effect estimates were indistinguishable from the null.

Several other studies investigated daily time-series data for time scales of exposure substantially longer than just a few days by using frequency domain log-linear regression (Dominici et al. 2003) or by using distributed lag models (Goodman et al. 2004; Zanobetti et al. 2003). Similar to these studies, we found larger RRs of mortality associated with particulate air pollution for longer time scales of exposure (15 days) than at time scales of a few days (6 days).

During times of drastic changes in concentrations of the exposures, in principle, two competing factors may explain a change in effect estimates. The first possibility is a nonlinear exposure–response relationship that converts to changes in the effect estimates over time as concentrations in the exposure change. The second possibility is a linear exposure–response relationship, but that the measured pollutants serve as indicators for changes in source composition or air pollution mixtures, so the associations with the outcome vary. We observed linear exposure–response functions and variations in RRs over time using time-varying coefficient models. However, we cannot fully exclude the possibility that we observed associations by chance due to the low statistical power.

Other studies have assessed whether changes in the levels or composition of the aerosol are associated with changes in health impacts (Clancy et al. 2002; Hedley et al. 2002). Laden et al. (2006) observed that mortality rates declined largest in cities with the largest reduction in long-term average PM using follow-up data from the Harvard Six Cities study. An analysis using data of the U.S. National Morbidity, Mortality, and Air Pollution Study showed only a weak indication that the association between PM_10_ and mortality declined during 1987–2000 and that this decline occurred mostly in the eastern United States (Dominici et al. 2007). A cross-sectional study conducted in three areas of Eastern Germany showed a decreasing prevalence of respiratory symptoms in schoolchildren along with declines of total suspended particulates and SO_2_ in the 1990s (Heinrich et al. 2002). Results from the present study suggest that the association between air pollution and mortality declined during 1995–2002. We observed the largest declines for the traffic-related air pollutants NO_2_, CO, and UFP, but we also observed a borderline significant decline for PM_2.5_. The biggest changes in mortality from period to period occurred when the local power plant was changed over from coal combustion to natural gas. Consequently, one may speculate whether the reduced chronic exposure to the coal combustion effluents may have contributed to the decline in daily mortality in the following few years.

### Strengths and limitations

Erfurt is a small city with < 5 deaths per day on average, which limits the statistical power of the analyses. However, because of the uniqueness of the long record of particle size distribution measures, it nevertheless serves as a natural experiment in which the impact of mobile source emissions, and of the transition from coal-based energy to modern technologies in energy generation, on ambient air pollution concentrations was recorded over a decade.

The use of a single monitoring site for the whole city of Erfurt may pose a limitation to this study. However, this site has been demonstrated to be representative for the air quality within the city of Erfurt with respect to PM_10_ and sulfate (Cyrys et al. 1998). One reason for the strong spatial correlation of the air pollutants in Erfurt is the geographic situation. Erfurt is confined with ridges on three sides and high-rise buildings on the fourth side. As a result, days with reduced air exchange rates between the city area of Erfurt and the surrounding rural area occur more frequently than in other German cities. Days with increased levels of ambient air pollutants are therefore more frequent than in other German cities.

Because UFP is mostly produced by local traffic, a greater spatial heterogeneity could be expected. However, concurrent measurements of UFP at different sites within one city often have shown good correlations despite differing magnitudes and suggest that a background site might well represent the exposure of the average population with respect to UFP if the site is carefully chosen (Aalto et al. 2005; Buzorius et al. 1999; Cyrys et al. 2008; Peters et al. 2005). Nevertheless, one would expect greater exposure misclassification by a single monitoring site for locally produced particles such as UFP than for regionally transported particles. Therefore, the fact that the present study did identify larger associations between locally generated particles and mortality than between PM_2.5_ or PM_10_ and mortality may point to an important role of locally produced particles on daily mortality.

Furthermore, the area of Erfurt, including the surrounding communities incorporated in 1994, is small. The doubling of the city region in the course of the administrative reform in 1994 may have led to less precise exposure assessment for the inhabitants of the newly incorporated communities, as suggested by the slightly larger risk estimates obtained for the old city center. However, about 90% of the inhabitants of Erfurt live within a rectangular area of 5 km × 3 km around the old city center.

We used a variety of pollutants for the analyses, because different pollutants may point toward differing properties of the aerosol and also represent different sources of air pollution. By testing a set of air pollutants, however, the possibility that some effects might have occurred by chance cannot be excluded. Because air pollutants are closely correlated, we considered especially consistent patterns in the data as actual effects. Moreover, we thoroughly adjusted for meteorologic confounder variables to rule out the possibility that the detected associations resulted from meteorologic influences or seasonal differences. In the present study, we smoothed confounding variables using penalized splines, instead of loess smoothers as applied by Wichmann et al. (2000), because of recent concerns about biased estimates of the standard errors due to concurvity when using loess smoothers in the statistical software package S-plus (Ramsay et al. 2003). The results are internally consistent and qualitatively agree with those from previous analyses over a shorter time period (Stölzel et al. 2003; Wichmann et al. 2000) with respect to UFP, despite the slightly different modeling strategy described above. The confidence limits for the RR estimates were somewhat smaller because of the larger amount of data. Additional sensitivity analyses indicated that our final model seemed to be conservative and stable with respect to the choice of the model parameters.

## Conclusions

Results indicate an elevated mortality risk from short-term exposure to UFP highlighting the potential importance of locally produced particles. The study further suggest that RRs for short-term associations of air pollution decreased as pollution concentrations decreased and control measures were implemented in Eastern Germany. The exposure–response functions were linear, and the concentration changes did not explain the variation in the coefficients.

## Figures and Tables

**Table 1:** Descriptive statistics of gaseous and particulate pollutants and meteorologic parameters in Erfurt, Germany, stratified for three periods.

Pollutant	Period	No.	Percent missing	Mean ± SD	Ratio of period means		
					1/3	1/2	2/3
NO_2_^a^ (μg/m^3^)	1	1,101	23.1	42.8 ± 24.9	1.46*	1.20*	1.21*
	2	901	1.2	35.5 ± 14.2			
	3	1,465	1.8	29.4 ± 11.8			
	Total	3,467	9.6	35.2 ± 18.5			

CO (mg/m^3^)	1	1,362	4.8	0.93 ± 0.65	2.67*	1.47*	1.82*
	2	912	0.0	0.64 ± 0.48			
	3	1,487	0.3	0.35 ± 0.23			
	Total	3,761	1.9	0.63 ± 0.54			

UFP^b^ (cm^−3^)	1^c^	165	88.5	13,198 ± 9,019	1.23*	0.80*	1.54*
	2	837	8.2	16,434 ± 10,468			
	3	1,357	9.1	10,702 ± 6,420			
	Total	2,359	38.5	12,910 ± 8,685			

PM_10_^b^ (μg/m^3^)	1	1,400	2.2	50.6 ± 32.2	2.08*	1.23*	1.69*
	2	912	0.0	41.1 ± 28.4			
	3	1,490	0.01	24.3 ± 15.4			
	Total	3,802	0.9	38.0 ± 28.3			

PM_2.5_^b^ (μg/m^3^)	1^c^	165	88.5	78.2 ± 78.6	4.50*	2.68*	1.68*
	2	899	1.4	29.2 ± 24.2			
	3	1,452	2.7	17.4 ± 12.3			
	Total	2,516	34.4	25.6 ± 30.4			

Air temperature (°C)	1	1,421	0.01	8.7 ± 7.5	0.97	1.27*	0.77*
	2	911	0.01	6.8 ± 8.1			
	3	1,491	0.01	8.9 ± 7.2			
	Total	3,823	0.01	8.3 ± 7.6			

Relative humidity (%)	1	1,421	0.01	77.6 ± 11.2	0.97*	0.95*	1.01*
	2	911	0.01	81.3 ± 11.0			
	3	1,491	0.01	80.2 ± 11.1			
	Total	3,823	0.01	79.5 ± 11.2			

Periods: period 1, 1 October 1991 to 31 August 1995; period 2, 1 September 1995 to 28 February 1998; and period 3, 1 March 1998 to 31 March 2002.

a Plausible data only; data from 1 April 1994 to 31 January 1995 omitted.

b Imputed time series.

c Measurements were scheduled only during the winter of 1991–1992.

* Statistically significant (*t*-test for independent variables, *p* < 0.05).

**Table 2:** Cumulative RRs of mortality per IQR increase of air pollutants in Erfurt, Germany, estimated with PDL models of lags up to 5 days with a third-degree polynomial, or estimated with means of lags 0–5.

			Cumulative 6-day RR (95% CI) per IQR	
Pollutant	Analysis period	6-day IQR	PDL model	Mean of lags 0–5
Old city limits				
NO_2_^a^ (μg/m^3^)	1991–2002	14.6	1.010 (0.988–1.031)	1.010 (0.990–1.030)
CO (mg/m^3^)	1991–2002	0.50	1.018 (0.990–1.046)	1.013 (0.987–1.039)
PM_10_^b^ (μg/m^3^)	1991–2002	24.2	0.997 (0.974–1.021)	0.998 (0.976–1.021)
New city limits				
NO_2_ (μg/m^3^)	1995–2002	12.7	1.001 (0.971–1.033)	1.008 (0.978–1.038)
CO (mg/m^3^)	1995–2002	0.31	1.009 (0.982–1.038)	1.014 (0.987–1.041)
PM_10_^b^ (μg/m^3^)	1995–2002	17.2	0.997 (0.972–1.022)	0.995 (0.971–1.019)
PM_2.5_^b^ (μg/m^3^)	1995–2002	13.3	1.009 (0.984–1.035)	1.004 (0.981–1.027)
UFP^b^ (cm^−3^)	1995–2002	8,439	1.041 (1.000–1.084)	1.032 (0.995–1.071)
NC 0.01–0.03 μm (cm^−3^)	1995–2001	5,765	1.034 (0.992–1.078)	1.024 (0.985–1.064)
NC 0.03–0.05 μm (cm^−3^)	1995–2001	1,783	1.033 (0.997–1.071)	1.029 (0.997–1.062)
NC 0.05–0.1 μm (cm^−3^)	1995–2001	1,008	1.026 (0.995–1.059)	1.022 (0.995–1.049)
Old city limits				
NO_2_ (μg/m^3^)	1995–2002	12.7	1.010 (0.978–1.043)	1.016 (0.985–1.048)
CO (mg/m^3^)	1995–2002	0.31	1.013 (0.984–1.043)	1.017 (0.989–1.047)
PM_10_^b^ (μg/m^3^)	1995–2002	17.2	1.004 (0.978–1.031)	1.001 (0.976–1.027)
PM_2.5_^b^ (μg/m^3^)	1995–2002	13.3	1.017 (0.990–1.044)	1.010 (0.986–1.035)
UFP^b^ (cm^−3^)	1995–2002	8,439	1.043 (1.000–1.088)	1.033 (0.994–1.073)
NC 0.01–0.03 μm (cm^−3^)	1995–2001	5,765	1.031 (0.987–1.078)	1.022 (0.981–1.064)
NC 0.03–0.05 μm (cm^−3^)	1995–2001	1,783	1.035 (0.996–1.074)	1.030 (0.996–1.065)
NC 0.05–0.1 μm (cm^−3^)	1995–2001	1,008	1.029 (0.996–1.064)	1.024 (0.995–1.053)

a Plausible data only; data from 1 April 1994 to 31 January 1995 omitted.

b Imputed time series.

**Table 3:** Cumulative RRs of mortality per IQR increase of air pollutants in Erfurt, Germany, estimated with PDL models of lags up to 14 days with a third-degree polynomial, or estimated with means of lags 0–14.

			Cumulative 15-day RR (95% CI) per IQR	
Pollutant	Analysis period	15-day IQR	PDL	Mean of lags 0–14
Old city limits				
NO_2_^a^ (μg/m^3^)	1991–2002	12.6	1.027 (1.000–1.055)	1.016 (0.995–1.038)
CO (mg/m^3^)	1991–2002	0.51	1.035 (0.995–1.077)	1.041 (1.007–1.075)
PM_10_^b^ (μg/m^3^)	1991–2002	22.3	1.021 (0.991–1.051)	1.020 (0.993–1.048)
New city limits				
NO_2_ (μg/m^3^)	1995–2002	11.0	1.017 (0.983–1.053)	1.012 (0.979–1.046)
CO (mg/m^3^)	1995–2002	0.31	1.021 (0.986–1.058)	1.021 (0.986–1.056)
PM_10_^b^ (μg/m^3^)	1995–2002	14.5	1.008 (0.982–1.036)	1.006 (0.981–1.032)
PM_2.5_^b^ (μg/m^3^)	1995–2002	11.5	1.019 (0.988–1.050)	1.017 (0.992–1.042)
UFP^b^ (cm^−3^)	1995–2002	7,649	1.060 (1.008–1.114)	1.055 (1.011–1.101)
NC 0.01–0.03 μm (cm^−3^)	1995–2001	5,336	1.051 (0.997–1.107)	1.045 (0.999–1.093)
NC 0.03–0.05 μm (cm^−3^)	1995–2001	1,483	1.045 (0.999–1.093)	1.034 (0.998–1.072)
NC 0.05–0.1 μm (cm^−3^)	1995–2001	805	1.032 (0.992–1.074)	1.019 (0.989–1.049)
Old city limits				
NO_2_ (μg/m^3^)	1995–2002	11.0	1.030 (0.993–1.068)	1.024 (0.989–1.060)
CO (mg/m^3^)	1995–2002	0.31	1.026 (0.988–1.064)	1.024 (0.988–1.062)
PM_10_^b^ (μg/m^3^)	1995–2002	14.5	1.019 (0.991–1.048)	1.017 (0.990–1.044)
PM_2.5_^b^ (μg/m^3^)	1995–2002	11.5	1.030 (0.997–1.063)	1.025 (0.999–1.052)
UFP^b^ (cm^−3^)	1995–2002	7,649	1.068 (1.013–1.126)	1.063 (1.016–1.111)
NC 0.01–0.03 μm (cm^−3^)	1995–2001	5,336	1.055 (0.998–1.114)	1.051 (1.003–1.102)
NC 0.03–0.05 μm (cm^−3^)	1995–2001	1,483	1.054 (1.005–1.105)	1.043 (1.005–1.082)
NC 0.05–0.1 μm (cm^−3^)	1995–2001	805	1.042 (0.999–1.087)	1.025 (0.994–1.057)

a Plausible data only; data from 1 April 1994 to 31 January 1995 omitted.

b Imputed time series.

**Table 4:** Cumulative RRs (95% CIs) of mortality per period in association with air pollution in Erfurt, Germany, estimated with multiday moving averages.

Pollutant	Overall IQR	Overall	Period 1	Period 2	Period 3
Cumulative 6-day RR per overall IQR					
NO_2_^a^ (μg/m^3^), old city limits	14.6	1.010 (0.990–1.030)	1.010 (0.987–1.033)	1.038 (0.990–1.089)	0.954 (0.907–1.003)
CO (mg/m^3^), old city limits	0.50	1.013 (0.987–1.039)	0.999 (0.971–1.028)	1.059 (1.007–1.114)	0.969 (0.888–1.057)
PM_10_^b^ (μg/m^3^), old city limits	24.2	0.998 (0.976–1.021)	0.996 (0.969–1.024)	1.013 (0.972–1.056)	0.949 (0.897–1.004)
PM_2.5_^b^ (μg/m^3^), new city limits	13.3	1.004 (0.981–1.027)		1.017 (0.990–1.044)	0.974 (0.937–1.013)
UFP^b^ (cm^−3^), new city limits	8,439	1.033 (0.996–1.071)		1.055 (1.011–1.105)	0.989 (0.932–1.049)
NC 0.01–0.03 μm^c^ (cm^−3^), new city limits	5,765	1.024 (0.985–1.064)		1.050 (1.000–1.102)	0.981 (0.924–1.043)
NC 0.03–0.05 μm^c^ (cm^−3^), new city limits	1,783	1.029 (0.997–1.062)		1.042 (1.005–1.081)	0.994 (0.939–1.052)
NC 0.05–0.1 μm^c^ (cm^−3^), new city limits	1,008	1.022 (0.995–1.049)		1.029 (0.998–1.061)	1.002 (0.954–1.052)
Cumulative 15-day RR per overall IQR					
NO_2_^a^ (μg/m^3^), old city limits	12.6	1.016 (0.995–1.038)	1.017 (0.993–1.042)	1.026 (0.974–1.081)	0.960 (0.905–1.019)
CO (mg/m^3^), old city limits	0.51	1.041 (1.007–1.075)	1.030 (0.993–1.068)	1.057 (0.989–1.130)	1.083 (0.967–1.213)
PM_10_^b^ (μg/m^3^), old city limits	22.3	1.020 (0.993–1.048)	1.017 (0.984–1.051)	1.012 (0.973–1.071)	0.978 (0.911–1.051)
PM_2.5_^b^ (μg/m^3^), new city limits	11.5	1.017 (0.992–1.042)		1.016 (0.988–1.045)	1.016 (0.971–1.063)
UFP^b^ (cm^−3^), new city limits	7,649	1.052 (1.009–1.097)		1.078 (1.023–1.136)	1.018 (0.948–1.093)
NC 0.01–0.03 μm^c^ (cm^−3^), new city limits	5,336	1.045 (0.999–1.093)		1.083 (1.022–1.148)	0.996 (0.926–1.070)
NC 0.03–0.05 μm^c^ (cm^−3^), new city limits	1,483	1.034 (0.998–1.072)		1.040 (0.998–1.083)	1.020 (0.954–1.091)
NC 0.05–0.1 μm^c^ (cm^−3^), new city limits	805	1.019 (0.989–1.049)		1.017 (0.984–1.052)	1.020 (0.964–1.082)

Period 1, 1 October 1991 to 31 August 1995; period 2, 1 September 1995 to 28 February 1998; and period 3, 1 March 1998 to 31 March 2002.

a Plausible data only; data from 1 April 1994 to 31 January 1995 omitted.

b Imputed time series.

c Only available between September 1995 and August 2001.

**Table 5:** Sensitivity analyses for UFP^a^ for the analysis period 1995–2002 in Erfurt, Germany.

	Cumulative 6-day RR (95% CI) per IQR (IQR = 8,439)		Cumulative 15-day RR (95% CI) per IQR (IQR = 7,649)	
Type	PDL model^b^	Mean of lags 0–5	PDL model^b^	Mean of lags 0–14
Original model	1.041 (1.000–1.084)	1.032 (0.995–1.071)	1.060 (1.008–1.114)	1.055 (1.011–1.101)
Trend with 6 df (fewer dfs than in the original model)	1.039 (0.999–1.081)	1.032 (0.996–1.070)	1.052 (1.002–1.104)	1.052 (1.009–1.096)
Air temperature variables with 6 df (more dfs than in the original model)	1.041 (1.000–1.084)	1.033 (0.996–1.072)	1.062 (1.010–1.117)	1.058 (1.014–1.104)
Indicator for day of the week (instead of Sunday indicator)	1.041 (1.000–1.084)	1.032 (0.995–1.071)	1.062 (1.010–1.117)	1.055 (1.011–1.101)
Old city limits and same confounder model as for the period 1991–2002	1.040 (0.997–1.085)	1.031 (0.992–1.071)	1.064 (1.010–1.122)	1.062 (1.016–1.110)

a Imputed time series.

b PDL models were estimated with a third-degree polynomial.

## References

[b1-ehp-117-448] Aalto P, Hameri K, Paatero P, Kulmala M, Bellander T, Berglind N (2005). Aerosol particle number concentration measurements in five European cities using TSI-3022 condensation particle counter over a three-year period during health effects pollution on susceptible subpopulations. J Air Waste Manage Assoc.

[b2-ehp-117-448] Acker K, Moller D, Marquardt W, Bruggemann E, Wieprecht W, Auel R (1998). Atmospheric research program for studying changing emission patterns after German unification. Atmos Environ.

[b3-ehp-117-448] AGI (German Influenza Working Group) (2003). Arbeitsgemeinschaft Influenza [in German].

[b4-ehp-117-448] Akaike H, Petrov BN, Csaki F (1973). Information theory and an extension of the maximum likelihood principle. Second International Symposium on Information Theory.

[b5-ehp-117-448] Analitis A, Katsouyanni K, Dimakopoulou K, Samoli E, Nikoloulopoulos AK, Petasakis Y (2006). Short-term effects of ambient particles on cardiovascular and respiratory mortality. Epidemiology.

[b6-ehp-117-448] Brezger A, Kneib T, Lang S (2005). BayesX: analyzing Bayesian structured additive regression models. J Stat Softw.

[b7-ehp-117-448] Brezger A, Lang S (2006). Generalized structured additive regression based on Bayesian P-splines. Comput Stat Data Anal.

[b8-ehp-117-448] Buzorius G, Hameri K, Pekkanen J, Kulmala M (1999). Spatial variation of aerosol number concentration in Helsinki city. Atmos Environ.

[b9-ehp-117-448] Clancy L, Goodman P, Sinclair H, Dockery DW (2002). Effect of air-pollution control on death rates in Dublin, Ireland: an intervention study. Lancet.

[b10-ehp-117-448] Cyrys J, Heinrich J, Brauer M, Wichmann HE (1998). Spatial variability of acidic aerosols, sulfate and PM10 in Erfurt, Eastern Germany. J Expo Sci Environ Epidemiol.

[b11-ehp-117-448] Cyrys J, Pitz M, Heinrich J, Wichmann HE, Peters A (2008). Spatial and temporal variation of particle number concentration in Augsburg, Germany. Sci Total Environ.

[b12-ehp-117-448] Dominici F, McDermott A, Daniels M, Zeger SL, Samet JM (2005). Revised analyses of the National Morbidity, Mortality, and Air Pollution Study: mortality among residents of 90 cities. J Toxicol Environ Health A.

[b13-ehp-117-448] Dominici F, McDermott A, Zeger SL, Samet JM (2003). Airborne particulate matter and mortality: timescale effects in four US cities. Am J Epidemiol.

[b14-ehp-117-448] Dominici F, Peng RD, Zeger SL, White RH, Samet JM (2007). Particulate air pollution and mortality in the United States: did the risks change from 1987 to 2000?. Am J Epidemiol.

[b15-ehp-117-448] Ebelt S, Brauer M, Cyrys J, Tuch T, Kreyling WG, Wichmann HE (2001). Air quality in postunification Erfurt, East Germany: associating changes in pollutant concentrations with changes in emissions. Environ Health Perspect.

[b16-ehp-117-448] Forastiere F, Stafoggia M, Picciotto S, Bellander T, D’Ippoliti D, Lanki T (2005). A case-crossover analysis of out-of-hospital coronary deaths and air pollution in Rome, Italy. Am J Respir Crit Care Med.

[b17-ehp-117-448] Friedman MS, Powell KE, Hutwagner L, Graham LM, Teague WG (2001). Impact of changes in transportation and commuting behaviors during the 1996 Summer Olympic Games in Atlanta on air quality and childhood asthma. JAMA.

[b18-ehp-117-448] Goodman PG, Dockery DW, Clancy L (2004). Cause-specific mortality and the extended effects of particulate pollution and temperature exposure. Environ Health Perspect.

[b19-ehp-117-448] Health Effects Institute (2003). Revised Analyses of Time-Series Studies of Air Pollution and Health.

[b20-ehp-117-448] Hedley AJ, Wong CM, Thach TQ, Ma S, Lam TH, Anderson HR (2002). Cardiorespiratory and all-cause mortality after restrictions on sulphur content of fuel in Hong Kong: an intervention study. Lancet.

[b21-ehp-117-448] Heinrich J, Hoelscher B, Frye C, Meyer I, Pitz M, Cyrys J (2002). Improved air quality in reunified Germany and decreases in respiratory symptoms. Epidemiology.

[b22-ehp-117-448] Laden F, Schwartz J, Speizer FE, Dockery DW (2006). Reduction in fine particulate air pollution and mortality: extended follow-up of the Harvard Six Cities study. Am J Respir Crit Care Med.

[b23-ehp-117-448] Lee D, Shaddick G (2007). Time-varying coefficient models for the analysis of air pollution and health outcome data. Biometrics.

[b24-ehp-117-448] Lee JT, Son JY, Cho YS (2007). Benefits of mitigated ambient air quality due to transportation control on childhood asthma hospitalization during the 2002 summer Asian games in Busan, Korea. J Air Waste Manage Assoc.

[b25-ehp-117-448] Peters A, Stölzel M, Cyrys J, Breitner S, Pitz M, Woelke G Improved air quality and its influences on short-term health effects in Erfurt, Eastern Germany. Res Rep Health Eff Inst.

[b26-ehp-117-448] Peters A, von Klot S, Heier M, Trentinaglia I, Cyrys J, Hormann A (2005). Particulate air pollution and nonfatal cardiac events. Part I. Air pollution, personal activities, and onset of myocardial infarction in a case-crossover study. Res Rep Health Eff Inst.

[b27-ehp-117-448] Pitz M, Kreyling WG, Holscher B, Cyrys J, Wichmann HE, Heinrich J (2001). Change of the ambient particle size distribution in East Germany between 1993 and 1999. Atmos Environ.

[b28-ehp-117-448] Pope CA, Dockery DW (2006). Health effects of fine particulate air pollution: lines that connect. J Air Waste Manage Assoc.

[b29-ehp-117-448] R Development Core Team (2003). R: A Language and Environment for Statistical Computing.

[b30-ehp-117-448] Ramsay TO, Burnett RT, Krewski D (2003). The effect of concurvity in generalized additive models linking mortality to ambient particulate matter. Epidemiology.

[b31-ehp-117-448] Samoli E, Aga E, Touloumi G, Nisiotis K, Forsberg B, Lefranc A (2006). Short-term effects of nitrogen dioxide on mortality: an analysis within the APHEA project. Eur Respir J.

[b32-ehp-117-448] Samoli E, Touloumi G, Schwartz J, Anderson HR, Schindler C, Forsberg B (2007). Short-term effects of carbon monoxide on mortality: an analysis within the APHEA project. Environ Health Perspect.

[b33-ehp-117-448] Schwartz J (2000). The distributed lag between air pollution and daily deaths. Epidemiology.

[b34-ehp-117-448] Stölzel M, Breitner S, Cyrys J, Pitz M, Wolke G, Kreyling W (2007). Daily mortality and particulate matter in different size classes in Erfurt, Germany. J Expo Sci Environ Epidemiol.

[b35-ehp-117-448] Stölzel M, Peters A, Wichmann HE (2003). Daily mortality and fine and ultrafine particles in Erfurt, Germany. Revised Analyses of Selected Time-Series Studies.

[b36-ehp-117-448] Touloumi G, Atkinson R, Le Tertre A, Samoli E, Schwartz J, Schindler C (2004). Analysis of health outcome time series data in epidemiological studies. Environmetrics.

[b37-ehp-117-448] Touloumi G, Samoli E, Pipikou M, Le Tertre A, Atkinson R, Katsouyanni K (2006). Seasonal confounding in air pollution and health time-series studies: effect on air pollution effect estimates. Stat Med.

[b38-ehp-117-448] Tuch T, Wehner B, Pitz M, Cyrys J, Heinrich J, Kreyling WG (2003). Long-term measurements of size-segregated ambient aerosol in two German cities located 100 km apart. Atmos Environ.

[b39-ehp-117-448] U.S. Environmental Protection Agency (2004). Air Quality Criteria for Particulate Matter.

[b40-ehp-117-448] Van Ness PH, Allore HG (2006). Using the SAS system to investigate effect modification.

[b41-ehp-117-448] WHO (1975). International Classification of Diseases.

[b42-ehp-117-448] WHO (1993). International Classification of Diseases.

[b43-ehp-117-448] WHO Europe (2006). Air Quality Guidelines. Global Update 2005. Particulate Matter, Ozone, Nitrogen Dioxide and Sulfur Dioxide.

[b44-ehp-117-448] Wichmann HE, Spix C, Tuch T, Woelke G, Peters A, Heinrich J (2000). Daily mortality and fine and ultrafine particles in Erfurt, Germany. Part I: role of particle number and particle mass. Res Rep Health Eff Inst.

[b45-ehp-117-448] Zanobetti A, Schwartz J, Samoli E, Gryparis A, Touloumi G, Peacock J (2003). The temporal pattern of respiratory and heart disease mortality in response to air pollution. Environ Health Perspect.

